# A novel, accurate, and non-invasive liquid biopsy test to measure cellular immune responses as a tool to diagnose early-stage lung cancer: a clinical trials study

**DOI:** 10.1186/s12931-023-02358-w

**Published:** 2023-02-14

**Authors:** Shafrira Shai, Fernando Patolsky, Hagai Drori, Eyal J. Scheinman, Eyal Davidovits, Giora Davidovits, Shoval Tirman, Nadir Arber, Amit Katz, Yochai Adir

**Affiliations:** 1 Savicell Diagnostics Ltd., Matam Advanced Technology Park, Building #23, P.O. Box 15050, 3190501 Haifa, Israel; 2 School of Chemistry, Faculty of Exact Sciences, Tel Aviv University, 69978 Tel Aviv, Israel; 3 Integrated Cancer Prevention Center, Tel Aviv Sourasky Medical Center, 6 Weizmann St., 6423906 Tel Aviv, Israel; 4 Department of General Thoracic Surgery, Rambam Health Care Campus, 8 HaAliya HaShniya St., PO Box 9602, 31096 Haifa, Israel; 5 Pulmonary Division, Faculty of Medicine, Lady Davis Carmel Medical Center, Mikhal Street 7, 3436212 Haifa, Israel

**Keywords:** Early stage, Biomarker, Liquid biopsy, Tumor associated antigens, Immune cells metabolism

## Abstract

**Introduction:**

Lung cancer remains the leading cause of death from cancer, worldwide. Developing early detection diagnostic methods, especially non-invasive methods, is a critical component to raising the overall survival rate and prognosis for lung cancer. The purpose of this study is to evaluate two protocols of a novel in vitro cellular immune response test to detect lung cancer. The test specifically quantifies the glycolysis metabolism pathway, which is a biomarker for the activation level of immune cells. It summarizes the results of two clinical trials, where each deploys a different protocol's version of this test for the detection of lung cancer. In the later clinical trial, an improved test protocol is applied.

**Method:**

The test platform is based on changes in the metabolic pathways of the immune cells following their activation by antigenic stimuli associated with Lung cancer. Peripheral Blood Mononuclear Cells are loaded on a multiwell plate together with various lung tumor associated antigens and a fluorescent probe that exhibits a pH-dependent absorption shift. The acidification process in the extracellular fluid is monitored by a commercial fluorescence plate reader device in continuous reading for 3 h at 37 °C to document the fluorescent signal received from each well.

**Results:**

In the later clinical trial, an improved test protocol was applied and resulted in increased test accuracy. Specificity of the test increased to 94.0% and test sensitivity increased to 97.3% in lung cancer stage I, by using the improved protocol.

**Conclusion:**

The improved protocol of the novel cellular immune metabolic response based test detects stage I and stage II of lung cancer with high specificity and sensitivity, with low material costs and fast results.

## Background

Lung cancer (LC) remains the worldwide leading cause of death from cancer. Unfortunately, approximately 75% of patients are diagnosed at an advanced stage of the disease (III, IV) [1]. Despite significant investment and advancement in LC research, only 16% of LCs are detected at the early stages [2]. Thus, even with recent advancements in treatment, survival remains poor. Developing early detection diagnostic methods, especially non-invasive methods, is a critical component in raising the overall survival rate and prognosis for lung cancer [3].

Current diagnostic methods (e.g., Computed Tomography—CT, Positron Emission Tomography—PET, Low-dose CT- LDCT, radiography) have high sensitivity but low specificity. False positive rates of 96.4% for LDCT and 94% for radiography [4–7] lead to a large number of unnecessary follow-up procedures. These procedures are expensive, invasive and can have significant complication risks. These can be pronounced in the elderly where para-physiological changes occur in the lungs which can lead to inappropriate interpretation of radiological findings that put patients at risk of over or under treatment as Baratella et al. report [8]. Recent work demonstrates that core-needle biopsy performed under CT leads to accurate histological diagnosis of LC with high sensitivity and specificity [9]. While it is less invasive than other procedures used to obtain tissue from the lung nodule, it is not without complication risks [9]. Invasive follow-up procedures are expensive and can have significant complication risks. Nuñez et al. reported [10] high frequency of complication rates, and factors associated with complications in a national sample of veterans screened for lung cancer by invasive procedures such as bronchoscopy, transthoracic needle lung biopsy and thoracic surgery. Shin et al. [11] demonstrated that after lung cancer surgery, pulmonary function and patient-reported outcomes noticeably decreased in the immediate postoperative period and improved thereafter, except for dyspnea and lack of energy. Hence, in recent years, several alternative liquid biopsy approaches such as metabolomic, transcriptomic, genomic, and proteomic [1, 12–16] for the identification of cancerous biomarkers have been explored for the early detection of LC. These approaches use different pathological, molecular, and biochemical analyses. Unlike invasive lung tissue biopsy to detect LC biomarkers, a liquid biopsy such as blood sample or other body fluid is non-invasive. For example, biomarkers as circulating cell-free tumor DNA (cf DNA), cell-free RNA (cf RNA), exosomes, tumor-educated platelets (TEP), and circulating tumor cells (CTCs) can be detected in blood to detect LC [17, 18]. Common to all of these diverse methods is that the detection of LC in its early stage has low sensitivity and/or specificity. Klein et al. validated a targeted methylation-based test to detect cancer and reported sensitivities of 16.8% to detect stage I and 40.4% to detect stage II [19, 20]. Xue et al. stated in their review of molecular technologies in liquid biopsy that early detection still needs to be improved [21].

Studies show that activation of immune cells requires changes in the way metabolic energy (ATP molecules) is generated. Immune system cells alter their energy generation in order to obtain an effector function. Usually, the shift is from the oxidative phosphorylation cycle into an aerobic glycolysis cycle. This shift provides immediate energy that gives the immune system the ability to attack the foreign antigen [22–24]. Hence, it appears that the activation state of the immune system, in response to tumor development, differs from the non-cancerous state [25–30]. These important discoveries corroborate our hypothesis that changes caused by cancer are reflected in different metabolic activity profiles of immune cells such as Peripheral Blood Mononuclear Cells (PBMCs) in response to various antigenic stimulants. In general, an effective in vitro response of the immune cells to re-stimulation with a LC tumor-associated antigen (TAA) stimulant indicates that the immune cells were previously exposed to the specific stimulant. Importantly, it indicates that the cells are able to produce an immunological response to it.

This article describes an improved immunometabolism blood test that measures the function of the immune cells in response to antigenic stimuli based on changes in the metabolic pathways of cells. There are several classical methods to test lymphocytes’ function. Mixed leukocyte culture (MLC) determines histocompatibility by co-culturing PBMCs of a potential donor with those of an allograft recipient. MLC takes 3–8 days to get results and involves the use of H^3^ thymidine radiolabeling [31]. Limiting dilution assay (LDA) also assesses histocompatibility between two parties. It determines the precursor frequencies of cytotoxic and helper T lymphocytes. The duration of this test is generally longer than MLC and takes 7–18 days [32]. Lymphocyte transformation test (LTT), in contrast to MLC and LDA, measures lymphocyte responses toward nonspecific stimuli (mitogens/drugs) or specific stimuli (antigen). A proliferative response shows that antigens of the respective microorganism are presented by antigen-presenting cells, and are recognized by pre-existing, antigen-specific T lymphocytes. The duration of this test is 8–10 days [33]. A more recent method to test the function of lymphocytes is the enzyme-linked immunospot (ELISpot) assay. It is a sensitive and quantitative method to detect cytokine production level in cell culture supernatant after growing cells with stimulant antigen. The duration of this ELISpot test, including cell culturing, is 2–12 days [34, 35]. Various flow cytometry assays that measure lymphocyte functionality include tests that are based on the detection of cell divisions by fluorescent CFSE staining, use of multimer staining of human leukocyte antigen (HLA) restricted peptides with their T cell receptor, use of other staining of cell’s receptors, or measurement of proteins that correlate with cell activation [36]. Like ELISpot, these types of tests need cell culturing for 2–12 days. ImmuKnow test measures the response of CD4^+^ T-helper lymphocytes to the mitogen phytohaemagglutinin-L (PHA), a general stimulator. It measures the amount of ATP produced by the cells following nonspecific stimulation. The duration of this test is 2 days [37]. While the methods described are non-invasive or devoid of the radiation risk of imaging, they all require days of execution, are cumbersome to perform, and there are no uniform standards (positive and negative controls, measurement units and working protocols) in performing these methods by different users. Therefore, the need for an assay that monitors in vitro cellular immune responses (primarily T and B cells) to antigenic stimuli with TAA, within a few hours, to determine immune activation levels is important.

In a previous publication, we presented a novel, non-invasive, cancer detection platform [38]. Our platform, named Liquid ImmunoBiopsy™, is based on measurements of metabolic activity profiles of immune cells. In our previous study we showed that by using machine learning methods to get a multivariate prediction model and training on the metabolic profiles, we were able to differentiate between blood samples of LC patients (n = 100, all stages) and control subjects (n = 100) with 91% sensitivity and 80% specificity in a cross-validation statistical evaluation*.* Since the clinical benefits for early detection of LC are demonstrated, we continued to develop the metabolic activity (MA) test protocol. The objective of this presented research is to investigate the accuracy of the metabolic activity test for lung cancer (MA-LC) in its improved protocol version versus the previous version by comparing MA-LC results from two additional clinical trials. The first clinical trial (n = 328) is referred to here as the “earlier” clinical trial, and the second additional clinical trial (n = 245) is referred to here as the “later” clinical trial. The earlier MA-LC protocol was used in the earlier clinical trial (n = 328), and an improved protocol was used in the later clinical trial (n = 245). We tested whether the improved protocol does, in fact, increase the sensitivity and specificity of the MA-LC to detect stage I and stage II LC.

## Methods

### Metabolic activity test

#### Improved protocol

Blood samples were collected in VACUETTE® tube 9 ml K3EDTA (Greiner Bio-One 455,036). Samples were transported in thermo-stated containers set to 18–25 °C until PBMCs separation. Fresh PBMCs were isolated by Lymphoprep™ kit, according to the manufacturer’s instructions (Axis-Shield). Total cell numbers were counted using automated cell counter LUNAII (Logos Biosystems). PBMCs concentration was adjusted to 4 × 10^6^ cells/ml with Assay Buffer PBS (Biological Industries Cat No 02-020-1A) with the addition of 5.5 mM glucose (Sigma Cat No G8769). Each well in a black non-binding, 96 multiwell plate (Greiner Bio-One) was loaded with 100 μl of the PBMCs solution and 100 μl of assay buffer containing 8-Hydroxypyrene-1,3,6-trisulfonic acid (HPTS, Thermo Fisher Cat No H348) in final concentration of 0.5 μM, and including 1 of the 8 TAA stimulants (short peptides 9–23 amino acids—Table 1) in final concentration of 10 μg/ml. These TAA were previously selected based on higher elicited metabolic shift reactivity of PBMCs of lung cancer patients than PBMCs of healthy subjects. The samples were loaded in triplicates and each triplicate appears twice in the plate layout, once in the right half side of the plate and once more in the left side. PBMCs were first loaded, followed by stimulants, to obtain a final volume of 200 μl in each well. Furthermore, each multiwell plate included four controls: one containing only the fluorescent HPTS probe without cells and without TAA stimulant; the second containing the HPTS probe with cells but without TAA stimulant, which represents the ‘basal state’; the third containing mitogen Phytohemagglutinin (PHA) in final concentration of 10 μg/ml, which is used as a positive control; and the fourth containing lactic acid in final concentration of 500 μM, used as chemical control. After the plate wells were loaded, the left half side of the plate was sealed hermetically with film (ThermalSeal RT™, Excel Scientific, Inc.) to avoid ventilation of CO_2_ and NH_3_, termed the ‘closed’ mode, while the right half side was left unsealed and termed the ‘open’ mode. Both states enable the measurement of real-time accumulation of ‘soluble’ versus ‘volatile’ metabolic products (lactic acid versus CO_2_ and NH_3_), thereby differentiating between oxidative phosphorylation (OXPHOS), anaerobic glycolysis and aerobic glycolysis. The multiwell plate was loaded into a commercial fluorescence plate reader device (filter based BioTek—Synergy H1 and Gen5 software ver. 3.11.19). Fluorescence intensities were measured at 513 nm under sequential excitation at wavelengths of 455 and 403 nm. The concentration of acidity units was calculated as a function of the ratio (d) between the two above-mentioned excitation wavelengths as described in our previous published article [38]. Acidification process was monitored in continuous reading for 3 h at 37 °C to document the fluorescent signal received from each well. These reflect time-dependent changes in acid concentration of the extracellular fluid in reaction to exposure to a stimulant or a control. The raw data from the plate reader device are processed instantly using proprietary Savicell software.

**Table 1:** List of TAA

Stimulant name	
New York esophageal squamous cell carcinoma-1 (NYESO-1)^a,b^	A human tumor antigen expressed in squamous cell carcinoma and adenocarcinoma
Melanoma-associated antigen A3 (MAGE-A3)^a,b^	Tumor antigens encoded by MAGE-A genes. Expressed in various tumor types but not in normal cells, except male germline cells or placenta
Gastrin-releasing peptide (GRP)^a^	A mitogenic molecule for many lung cell types. GRP peptides bind to specific surface receptors and initiate a complex cascade of signaling events (including MAPK and EGFR involvement) that culminates in the stimulation of DNA synthesis and cancer cell division
Human Epidermal Growth Factor Receptor 2 (HER2)^a,b^	A transmembrane glycoprotein and member of the epidermal growth factor receptor family. HER2 deregulation, including overexpression, amplification, and mutation, has been described in NSCLC
Neuron-specific enolase (NSE)^a^	A dimeric isoform of the glycolytic enzyme enolase found mainly in neurons. A well-known marker of small cell lung cancer and for NSCLC
Phytohemagglutinin (PHA)	A mitogen that induces non-specific activation of T/B immune cells

^a^Proteins in which only a partial sequence of ~ 9–23 amino acids were used

^b^Two such partial sequences were used

#### Previous protocol

A description of the assay can be found in a previously published article [38]. In brief, each well in a black non-binding, low-volume 384 multiwell plate (Greiner Bio-One) was loaded with 10 μl of the PBMCs solution (5 × 10^6^ cells/ml) and 10 μl of 10 mM PBS containing 1 of the 16 stimulating reagents (stimulants) in increasing concentrations and 0.5 μM/well HPTS. Each test plate included two controls: one containing only the fluorescent HPTS probe, without cells and without stimulants; the other containing the HPTS probe with cells but without stimulants, which represents the ‘basal state’. The acidification process was monitored for approximately 1.5 h at 37 °C by a commercial fluorescence plate reader (TECAN Infinite M200/ F200; application Tecan i-control 1.10.4.0, 1.11.1.0, 1.12.4.0). First, the reader monitored the acidification process without a plate seal (‘open’ state), and then, the multi-well plate was sealed hermetically (ThermalSeal RT™, Excel Scientific, Inc.) to avoid ventilation of CO_2_ and NH_3_ for the second phase of the test (‘closed’ state).

### Cohort details of the earlier clinical trial

**Fig. 1:**
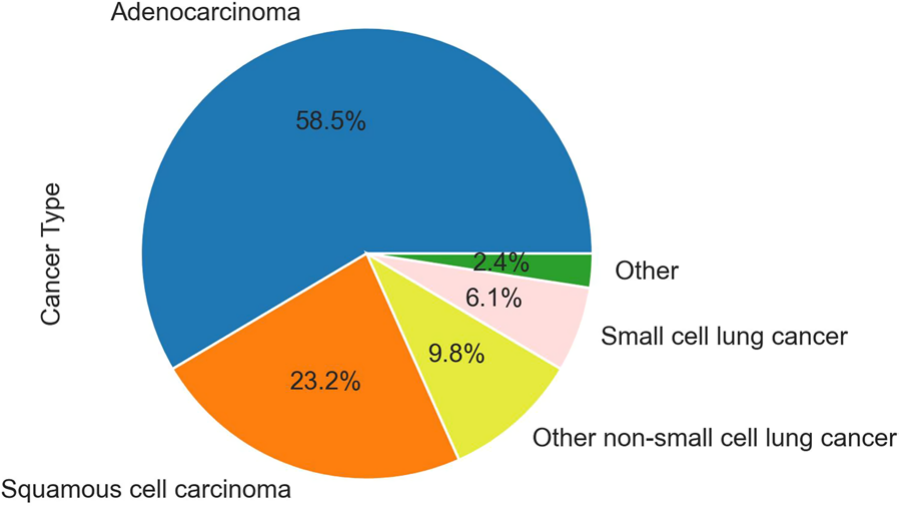
Lung cancer type distribution. Distribution of lung cancer types in the cohort of the earlier clinical trial. Subjects were enrolled between 2014-06-02 and 2017-11-16 in three medical centers. The 'Other' group includes lung cancer types other than non-small cells lung cancer and small cells lung cancer

Subjects were enrolled between 2014-06-02 and 2017-11-16 in three medical centers. In all cases, the study received approval of Institutional Review Boards (IRB) in accordance with the Declaration of Helsinki, and subjects read and signed a dedicated consent form. Inclusion and exclusion criteria were applied as described in our previously published article [38], apart from excluding lung cancer stages III and IV from the cohort. The reference standard for lung cancer is biopsy or surgery and the cancer stage is determined by a physician specialist based on defined medical criteria. Control subjects were age and sex matched with lung cancer subjects (Fig. 1).

### Cohort details of the later clinical trial

**Fig. 2:**
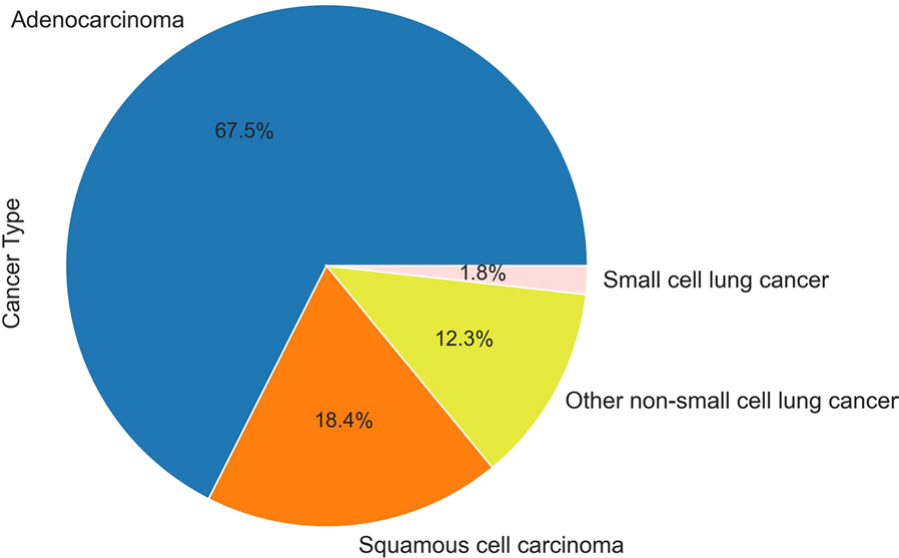
Lung cancer type distribution. Distribution of lung cancer types in the cohort of the later clinical trial. Subjects were enrolled between 2019-03-24 and 2021-03-09 in three medical centers

Subjects were enrolled between 2019-03-24 and 2021-03-09 in three medical centers. In all cases, the study received IRB approval in accordance with Declaration of Helsinki, and subjects read and signed a dedicated consent form. The same inclusion and exclusion criteria were applied as described in our previous published article [38] apart from that subjects with diabetes were included in this trial as we previously learned that diabetes does not impact the MA-LC results. The reference standard for lung cancer is biopsy or surgery and the cancer stage is determined by a physician specialist based on defined medical criteria. Control subjects were age and sex similar with lung cancer subjects. Subjects with different types of lung cancer and different stages of lung cancer were included, with emphasis on early stages for cross validation statistical evaluation (Fig. 2).

### Data analysis

Each subject was assigned a datasheet containing raw fluorescent readings of the plate wells as a function of time. The fluorescent readings were transformed into values which correlate with the acidity of the sample. We modeled the biological progression of the immunological response and extracted a set of informative features for use in our assay.

Machine Learning (ML) models were trained using logistic regression implemented by the publicly available scikit-learn Python library. For cross-validation, a stratified fivefold split was used. Confidence interval (CI) was calculated using Wilson score interval. Each stage is displayed separately for sensitivity calculation. We determined the decision boundary by choosing the point on the ROC curve with the highest Euclidean distance from the main diagonal. For a measure of separation between lung cancer and control subjects, the area under the receiver operating characteristic curve (AUC) was chosen. Repeated evaluations with different random, cross validation train/test splits were performed to verify result stability. Our cancer reference method is based on the results from tissue biopsy pathology of lung nodules, which determines whether there is a malignancy, and the lung cancer type. The cancer stage is determined by a physician specialist based on defined medical criteria.

## Results

### Analytical sensitivity and specificity of the metabolic activity lung cancer test (MA-LC)

The Metabolic Activity test measures the change in acid concentration over time in reaction to exposure to a stimulant. We use 8-Hydroxypyrene-1,3,6-trisulfonic acid (HPTS)—a highly water-soluble, membrane-impermeant pH indicator (pH 6.6 to 8.0) that is added to each plate’s well. HPTS exhibits a pH-dependent adsorption shift that allows the performance of ratiometric pH measurements by using the excitation ratio of 403/455 nm (d) that correlates with acidity. Analytical sensitivity of the newly developed metabolic activity test represents the smallest amount of change over time of acidity in a sample that can be accurately measured by the MA test.

To calculate the analytical sensitivity, we first determined the limit of blank (LOB). Our blank is PBMCs without a stimulant. We measure the highest result that is likely to be observed with a blank with certainty of 90% (z-score of 1.645 times the standard deviation of the repeats). Next, we’ve added to the LOB the lowest measurement result that is likely to be observed with PBMCs with a stimulant with certainty of 90%. The calculated lower limit of quantification (LLOQ) for metabolism activity based on the above calculations is 0.000119 (d/minute) in change of acidity over time. This points out the high sensitivity of the MA test to detect tiny changes in acid concentration over time.

**Fig. 3:**
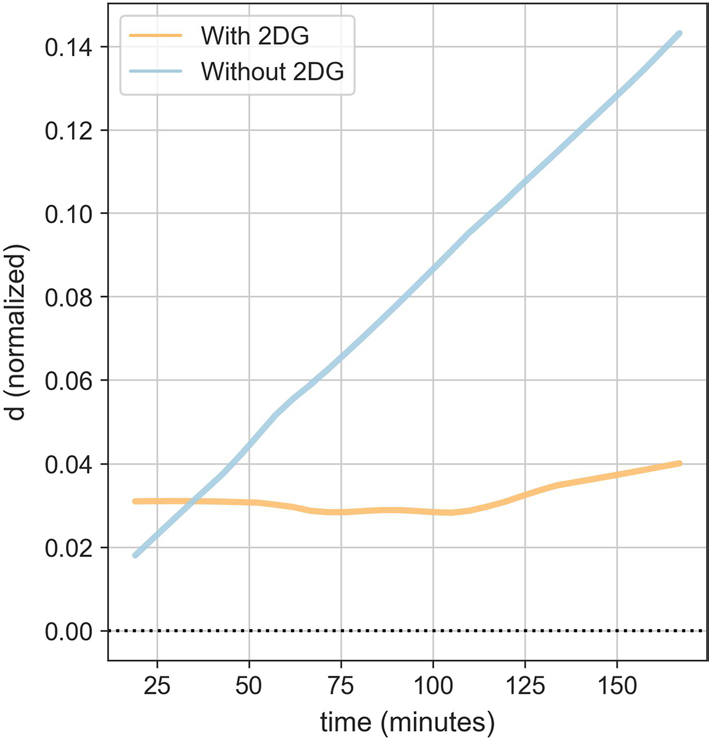
Acidity signal is related to glycolysis metabolic pathway (2DG suppresses acidification/glycolysis). The blue line shows acidity (d) changes over time of PBMCs together with a general stimulant (mitogen PHA). The orange line shows acidity (d) changes over time of PBMCs together with a general stimulant (mitogen PHA) and 2DG (10 mM/well)

The MA test measures time-dependent changes in acid concentration of the extracellular fluid in a reaction that relates to the glycolysis metabolic cycle. We verified that the test specifically quantifies glycolysis by measuring the acidity (d) change over time. We use 2-deoxy-D-glucose (2-DG), a glucose analogue able to suppress glycolysis by competitively inhibiting hexokinase 2 (HK2). Adding a general stimulant (mitogen PHA, stimulates metabolic activity in a nonspecific manner) to PBMCs causes detectable extracellular acidification reaction resulting from the secretion of lactic acid, a product of the glycolysis pathway into the extracellular fluid (blue line—Fig. 3) while adding 2DG (10 mM/well) together with PHA to PBMCs (using PBMCs from the same subject), prevented the extracellular acidification as a result of the inhibition effect of 2DG on glycolysis metabolic pathway (orange line—Fig. 3).

**Fig. 4:**
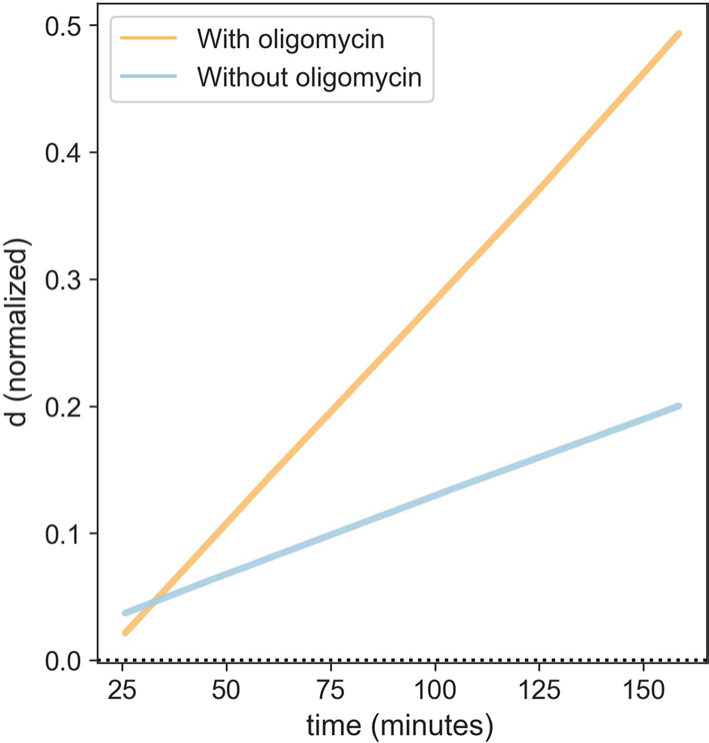
Inhibition of oxidative phosphorylation metabolic pathway by OMC increases glycolysis. LThe orange line shows acidity (d) changes over time of PBMCs together with a general stimulant (mitogen PHA) and OMC. The blue line shows acidity (d) changes over time of PBMCs together with a general stimulant (mitogen PHA)

Oligomycin (OMC) is an inhibitor of the oxidative phosphorylation metabolic cycle. It is an Adenosine Triphosphate (ATP) synthase inhibitor that prevents phosphorylation of Adenosine Diphosphate (ADP) to ATP. Inhibition of ATP synthase via the oxidative phosphorylation metabolic cycle stimulates the increase of the glycolysis cycle in order to meet the energy production need of the cell. We verified that adding OMC (4 μM/well) together with PHA to PBMCs increased the glycolysis rate (orange line—Fig. 4) compared to the glycolysis rate that was detectable in PBMCs with PHA alone (blue line—Fig. 4). The test specifically quantifies the increase in glycolysis metabolic pathway caused by OMC inhibition on the oxidative phosphorylation metabolic cycle.

These two experiments show that acidity changes over time in PBMCs extracellular fluid correlate with changes of the glycolysis metabolic cycle.

### Data analysis

Each subject was assigned a datasheet containing raw fluorescent readings of the plate wells as a function of time. The fluorescent readings were transformed into values which correlate with the acidity of the sample. We modeled the biological progression of the immunological response and extracted a set of informative features for use in our assay. Machine Learning (ML) models were trained using logistic regression implemented by the publicly available scikit-learn Python library. For cross-validation, a stratified fivefold split was used. We determined the decision boundary by choosing the point on the receiver operating characteristic (ROC) curve with the highest Euclidean distance from the main diagonal. Repeated evaluations with different random, cross validation train/test splits were performed to verify result stability. Our cancer reference method is based on the results from tissue biopsy pathology of lung nodules, which determines whether there is a malignancy, and the lung cancer type. The cancer stage is determined by a physician specialist based on defined medical criteria.

### Sensitivity and specificity of MA-LC for detection early-stage LC of the earlier clinical trial

**Fig. 5:**
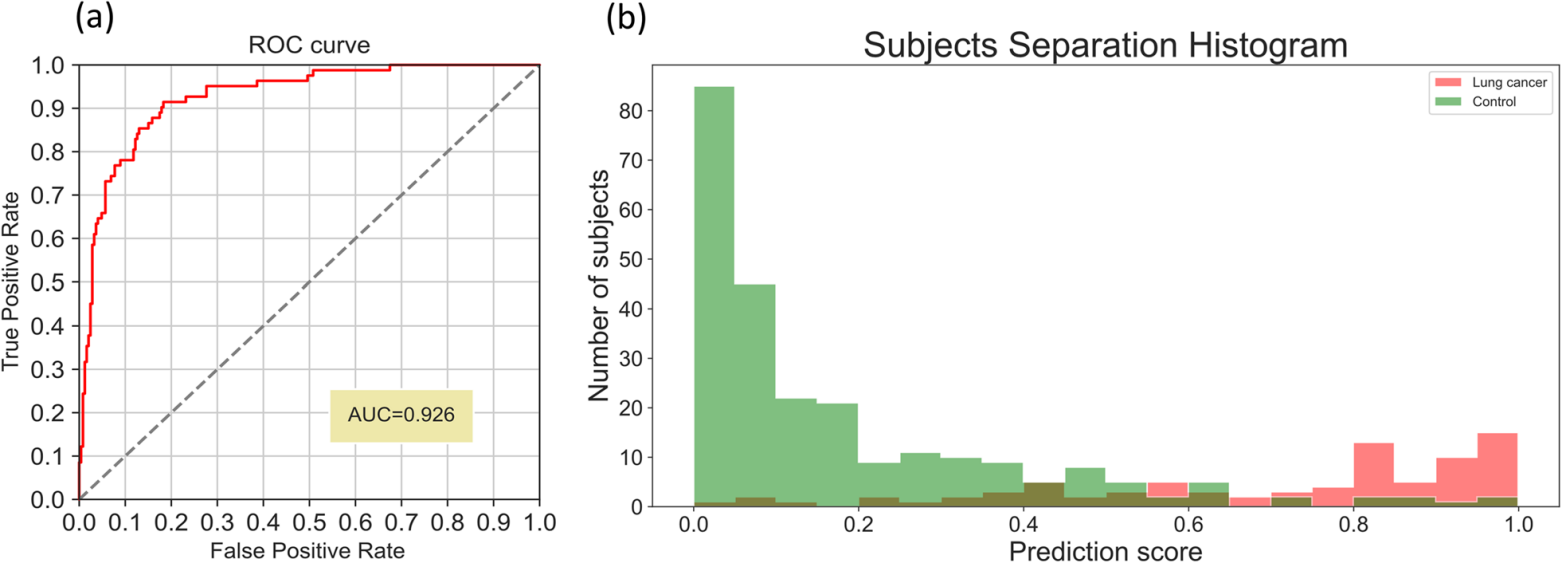
Receiver operating characteristic (ROC) curve (**a**) and a histogram of prediction scores (**b**), visual of the separation between lung cancer and control subjects—the earlier clinical trial. **a** Shows receiver operating characteristic (ROC) curve; random changes for detecting cancer are plotted with a dashed line. **b** Shows a histogram of prediction scores; ranges of prediction scores that contain both cancer and control subjects have overlapping bars

Performance was evaluated using a stratified fivefold cross validation. Confidence interval (CI) was calculated using Wilson score interval. Clinical stages I and II are displayed separately for sensitivity calculation; for a measure of separation between lung cancer and control subjects, the area under the receiver operating characteristic curve (AUC) was chosen (Fig. 5). Table 2 shows demographics and clinical characteristics for participating subjects in the earlier clinical trial.

**Table 2:** Demographics and clinical characteristics for participating subjects (n = 328) – earlier clinical trial

Characteristic	Lung cancer group (n = 82)	Control group (n = 246)	All (n = 328)
Age (years)^a^	66.7 ± 9.6	65.0 ± 8.1	65.4 ± 8.5
Sex			
Male	46	137	183
Female	36	109	145
Smokers			
Current	29	41	70
Former^b^	34	89	123
Clinical stage			
I	63	–	–
II	19	–	–
Histological type			
Adenocarcinoma	48	–	–
Squamous cell carcinoma	19	–	–
Other non-small cell	8		
Small cell	2		
Other	5	–	–

^a^The age of subjects at blood withdrawal

^b^Subjects with at least one pack-year in their history, who have not smoked in the past 30 days

### Sensitivity and specificity of MA-LC for detection early-stage LC of the later clinical trial

**Fig. 6:**
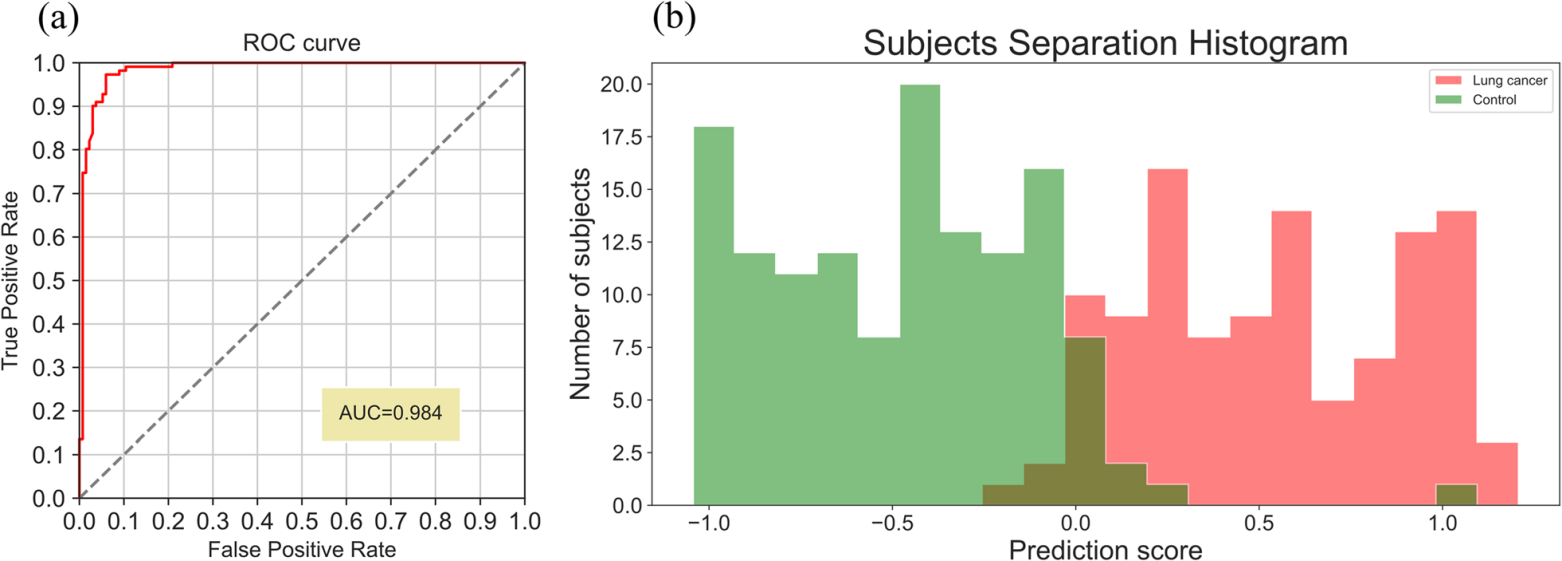
Receiver operating characteristic (ROC) curve (**a**) and a histogram of prediction scores (**b**); visual of the separation between lung cancer and control subjects—the later clinical trial. **a**) Shows receiver operating characteristic (ROC) curve; random changes for detecting cancer are plotted with a dashed line. **b** Shows a histogram of prediction scores; ranges of prediction scores that contain both cancer and control subjects have overlapping bars

Performance was evaluated using a stratified fivefold cross validation. Confidence interval (CI) was calculated using Wilson score interval. Clinical stages I, II and III, IV were combined for sensitivity calculation; N/A indicates cases where the cancer clinical stage was not available. For a measure of separation between lung cancer and control subjects, the area under the receiver operating characteristic curve (AUC) was chosen (Fig. 6). Table 3 shows demographics and clinical characteristics for participating subjects in the later clinical trial.

**Table 3:** Demographics and clinical characteristics for participating subjects (n = 245) – later clinical trial

Characteristic	Lung cancer group (n = 111)	Control group (n = 134)	All (n = 245)
Age (years)^a^	67.9 ± 8.8	52.4 ± 13.5	59.4 ± 13.9
Sex			
Male	74	73	147
Female	37	61	98
Smokers			
Current	61	47	108
Former^b^	30	18	48
Clinical stage			
I	39	–	–
II	21	–	–
III	25	–	–
IV	25	–	–
N/A	1		
Histological type			
Adenocarcinoma	77	–	–
Squamous cell carcinoma	21	–	–
Other	13	–	–

^a^The age of subjects at blood withdrawal

^b^Subjects with at least one pack-year in their history, who have not smoked in the past 30 days

## Discussion

We describe an improved immunometabolism blood test that measures the function of the immune cells in response to LC antigenic stimuli based on enhancement of the glycolysis metabolic pathway of immune cells. Glycolysis enhancement is a marker for the rapid activation of most immune cells [29].

This research article compares results from two clinical trials; in the earlier clinical trial an earlier MA-LC protocol was used and in the later clinical trial an improved protocol was used. Since the clinical benefits for early detection have been demonstrated, this current research focuses on early-stage lung cancer (stages I, II). Our results indicate that the MA-LC in its final version improves the test’s specificity from 81.7% (Table 4 – earlier clinical trial) to 94% (Table 5 – later clinical trial), while sensitivity increased from 92.3% (Table 4 – earlier clinical trial) to 94.9% (Table 5 – later clinical trial) in identifying LC stage I, and from 89.5% (Table 4 – earlier clinical trial) to 100% (Table 5 – later clinical trial), in identifying LC stage II. The higher specificity and sensitivity in the later clinical trial is the result of fine tuning the previously published protocol. These improvements include calibration of stimulants and PBMCs concentrations, selection of the most suitable stimulants, and improvements in quality control methods.

**Table 4:** Sensitivity and specificity of MA-LC of the earlier clinical trial

Performance measures		CI (95%)
Specificity	81.7%	(76–86%)
Sensitivity	91.5%	(83–96%)

Sensitivity per stage		
Stage	Number of subjects	Sensitivity
I	63	92.3%
II	19	89.5%

**Table 5:** Sensitivity and specificity of MA-LC of the later clinical trial

Performance measures		CI (95%)
Specificity	94.0%	(89–97%)
Sensitivity	97.3%	(92–99%)

Sensitivity per stage		
Stage	Number of subjects	Sensitivity
I	39	94.9%
II	21	100.0%
III	25	100.0%
IV	25	96.0%
N/A	1	100.0%

The sensitivity and specificity obtained by MA-LC in detecting early-stage lung cancer is much higher than the results reported for stages I, II in the literature by using only one method [19–21, 39]. The superior accuracy for early stages by MA-LC can be explained by the hypothesis that immune cells in lung-associated lymph nodes reach the malignant cells in the lung when the tumor is young, small and has yet to develop its ability to evade immune cells (stages I, II). Recognition of lung cancer TAAs (stimulants) by immune cells is possible and results in an immediate shift to the glycolysis pathway, enabling an effective local immune response. As the malignant tumor develops and grows (cancer at later stages), it activates mechanisms for evading the immune system. This results in the failure of the immune system to adequately activate and allows the tumor to escape immune detection and elimination [40]. Tumor-exposed immune cells reach peripheral blood, and repeated in vitro exposure to TAAs will result in a shift to the glycolysis metabolic pathway. This shift is detectable by the MA-LC. Metabolic pathway shift and immune cell function are highly correlated [29]. For example, the activation of immune receptors promotes glycolysis, which is the energy source of immune cells to fight the foreign invader tumor antigens.

Other biomarkers noninvasive tests focus on detecting circulating biomarkers, including tumor DNA, tumor antigens, tumor cells, exosomes, and extracellular vesicles. These biomarkers are released to peripheral blood primarily when the tumor reaches a certain size at a later stage of the disease.

We have previously shown [38] that chronic obstructive pulmonary disease (COPD) or smoking habits do not affect the test results, which supports other findings that diseases produce specific signatures in the metabolic profiles, which can help distinguish between various ailments such as cancers, autoimmune diseases, and infectious diseases [23]. To date, there are no laboratory tests for lymphocyte function that provide a quick and accurate answer. The immunometabolism assay can help diagnose early stages of cancer by using tumor associated/specific peptides.

The broad potential of this immunometabolism-based platform may also extend to other types of diseases, as well as to treatment monitoring and therapy selection. It could provide the cellular immune status of vaccinated people to SARS-COV-2 by using virus spike peptides, measure other cellular immune statuses to diseases such as allergy, autoimmune, immunodeficiency, antimicrobial immunity, follow up the effect of immunotherapy treatments, and measure drug efficacy.

Liquid ImmunoBiopsy™ is a new, promising, and non-invasive platform that measures the metabolic state of the immune system as a direct indicator of cellular immune responses (primarily T and B cells) to antigenic stimuli. The MA-LC provides results within five hours of receiving the blood sample for MA-LC. The analytical sensitivity of the test is high with a lower limit of quantification (LLOQ) of 0.000119 (d/minute) in change of acidity over time. It specifically quantifies glycolysis which is a biomarker for the activation level of immune cells that are re-exposed in vitro to lung TAA stimulants.

The present study has limitations. Not all control subjects received LDCT screening, nor were they followed-up after the blood draw, therefore, it is unknown whether lung cancer cases were already present and missed. Separately, cross validation is a widely used approach for assessment of classification performance and can address known individual confounders. However, cross-validation procedures do not control simultaneously for all confounders, and the use of an independent test set is needed to evaluate the generalizability of these results. Prospective studies are planned to validate classifier performance in an independent cohort and verify the generalization predictions from confounder-controlled CV.

The MA-LC is, *inter alia*, a diagnostic method to detect stage I and stage II of lung cancer, with low material costs and fast results. Furthermore, the combination of LDCT scans with MA-LC may reduce the need for follow-ups of suspected lung nodules, prevent unnecessary radiation exposure, and decrease the number of unnecessary invasive procedures with their associated complications. In addition, the MA-LC can help improve adherence to routine medical screenings in high-risk populations through the use of a patient-friendly blood test. A larger prospective clinical validation is a next step.

## Conclusions

Data analysis of a clinical trial applying the improved protocol of the ImmunoBiopsy™ test shows test specificity and sensitivity of 94.0% and 97.3%, respectively in detecting lung cancer stage I, and test specificity and sensitivity of 94.0% and 100%, respectively in detecting lung cancer stage II. The sensitivity and specificity obtained by this test in detecting stage I and stage II lung cancer is significantly higher than the results reported for stage I and stage II in the literature that uses only one method. ImmunoBiopsy™ is a promising, and non-invasive test to help diagnose early stages of lung cancer with low material costs and fast results. Detecting lung cancer in its early stage is a critical component in raising the overall survival rate and prognosis for lung cancer.

## Data Availability

Available from the corresponding author on reasonable request.

## Abbreviations

LC: Lung cancer
CT: Computed tomography
PET: Positron emission tomography
LDCT: Low-dose CT
cfDNA: Circulating cell-free tumor DNA
cfRNA: Cell-free RNA
TEP: Tumor-educated platelets
CTCs: Circulating tumor cells
PBMCs: Peripheral Blood Mononuclear Cells
TAA: Tumor-associated antigen
MLC: Mixed leukocyte culture
LDA: Limiting dilution assay
LTT: Lymphocyte transformation test
ELISpot: Enzyme-linked immunospot
HLA: Human leukocyte antigen
PHA: Phytohaemagglutinin-L
MA: Metabolic activity
MA-LC: Metabolic activity test for lung cancer
HPTS: 8-Hydroxypyrene-1,3,6-trisulfonic acid
OXPHOS: Oxidative phosphorylation
IRB: Institutional Review Boards
LOB: Limit of blank
LLOQ: Lower limit of quantification
2-DG: 2-Deoxy-d-glucose
OMC: Oligomycin
ATP: Adenosine Triphosphate
ADP: Adenosine Diphosphate
ML: Machine Learning
ROC: Receiver operating characteristic
CI: Confidence interval
AUC: Area under the receiver operating characteristic curve
COPD: Chronic obstructive pulmonary disease
